# Use of Global Risk Score for Cardiovascular Evaluation of Rural Workers in Southern Brazil

**DOI:** 10.1155/2018/3818065

**Published:** 2018-03-18

**Authors:** Marta Regina Cezar-Vaz, Clarice Alves Bonow, Marlise Capa Verde Almeida de Mello, Daiani Modernel Xavier, Jordana Cezar Vaz, Maria Denise Schimith

**Affiliations:** ^1^School of Nursing, Federal University of Rio Grande, 96203-900 Rio Grande, RS, Brazil; ^2^Faculty of Nursing, Federal University of Pelotas, Pelotas, RS, Brazil; ^3^Medicine Course, UNIFENAS, Alfenas, MG, Brazil; ^4^Department of Nursing, Federal University of Santa Maria, Santa Maria, RS, Brazil

## Abstract

The objectives of the present study were to classify the cardiovascular evaluation of rural workers through the Global Risk Score and analyze the intensity of the relationship between the classification and the variables of the score. A descriptive study was developed with 38 rural workers from the extreme southern Brazil. Data collection was performed through an interview and verification of anthropometric measurements and arterial blood pressure. Data were analyzed descriptively and inferentially using the Spearman correlation test. The overall 10-year cardiovascular risk classification using the Framingham global score showed a predominance of low risk (*n* = 22; 57.9%); however, 11 rural workers (28.9%) had a high cardiovascular risk. Spearman's correlation analysis showed significance between the Global Risk Score and gender (rho = 0.623, *p* ≤ 0.001), age (rho = 0.783, *p* ≤ 0.001), systolic blood pressure (rho = 0.545, *p* ≤ 0.001), and smoking (rho = 0.483, *p* = 0.002). These results show that rural workers need attention with regard to components that may put them at risk for cardiovascular disease. This study may help in the early diagnosis and more effective actions on the risk factors for cardiovascular disease.

## 1. Introduction

Cardiovascular diseases (CVD) represent a global public health problem. However, it is known that these diseases are preventable through reduction of behavioral risk factors, such as tobacco use, inadequate diet (rich in salt, fat, and calories) and physical inactivity [1]. In this sense, the Brazilian situation is of concern since in a survey conducted by phone, it was indicated that 25.7% of the people reported a diagnosis of hypertension, being higher in women (27.5%) than in men (23.6%), 8.9% reported diabetes, with 7.8% men and 9.9% women, and 22.6% indicated dyslipidemia, being higher among females (25.9%) than among males (18.8%) [2].

In order to identify common risk factors that contribute to these diseases, the Framingham Heart Study developed a score that stratifies cardiovascular risk, estimating CVD involvement in 10 years [3]. Widely used all over the world it has been applied in studies conducted in the United States [4], Africa [5], Brazil [6], some European countries [7, 8], and Asia [9, 10]. From this score, others were developed, such as the Global Risk Score (GRS), adapted to Primary Health Care [11]. Some studies show that, over time, the risk of individuals presenting CVD is increasing [5, 10], which shows a lack of adherence to reduce risk factors.

In this sense, it can be estimated that in the urban environment there may be greater indications of a lifestyle that favors the occurrence of risk for CVD, such as high salt, sugar, and fat consumption and sedentary lifestyle. However, a study carried out in Mexico on the prevalence of cardiovascular risk in rural, suburban, and urban populations showed that suburban and rural populations presented greater risk in relation to urban [12]. One of the risks for developing CVD presented by the rural population in this study was excess abdominal fat [12], which contributes greatly to the development of the disease [13].

It is relevant to study the rural workers, who carry out unhealthy activities, such as animal milking [14] and who, due to work, live in distant and difficult places [15]. In this way, this study presents to classify the cardiovascular evaluation of rural workers through the Global Risk Score and analyze the intensity of the relationship between the classification and the variables of the score.

## 2. Methods

This is a transversal, exploratory, and descriptive study carried out with rural workers from the extreme south of Brazil. The nonprobabilistic sample, for convenience, consisted of 38 rural workers who carried out activities of agriculture of fruits and vegetables (lettuce, arugula, orange, and strawberry) and animal milking, presenting a minimum age of 30 years and a maximum of 74 years, since the GRS is applied to individuals in this age group [11], and had no previous history of CVD.

To collect data, a questionnaire was built by the research group, which had socioeconomic issues (skin color/ethnicity, marital status, schooling, and rural working time) and specific to GRS application (gender, age, value of systolic blood pressure, treatment for hypertension, smoking, diagnosis of diabetes, and body mass index (BMI)). The diagnosis of hypertension and diabetes was self-reported by study participants.

For the estimation of the GRS, it was chosen to replace the laboratory clinical examination about the lipid profile of the individuals by BMI [11], since most of the interviewees did not have a laboratory examination that included total cholesterol and HDL cholesterol in the last 12 months.

The BMI was measured after verifying the anthropometric measures of the participants (weight and height). The verification of the measures occurred at the time of questionnaire application. Weight and height were measured using an anthropometric balance (Welmy, with 100 g precision). BMI can be used to classify underweight (<18.50), overweight (≥25.00), and obesity (≥30.00), divided between obesity class I (between 30.00 and 34.99), obesity class II (35.00 and 39.99), and obesity class III (≥40.00) [16].

The determination of systolic blood pressure was performed using an aneroid sphygmomanometer (Premium®), tested, and calibrated. The measurement was performed in the sitting position, with the right arm resting on a table.

After data collection, data were entered into an electronic calculator available on the Framingham Heart Study website [3, 11]. The data insertion in the calculator provides a percentage, which rates the individual's CVD risk over a 10-year period. The classification is stratified as low risk <6%, moderate risk 6% to 20%, and high risk > 20% [11].

The data were quantified in the software IBM Statistical Package for the Social Sciences, version 19.0. The variables were described by mean, standard deviation, and absolute frequency. The Spearman correlation was performed to analyze the intensity of the relationship between the GRS and the variables that compose it (gender, age, value of systolic blood pressure, treatment for hypertension, smoking, diagnosis of diabetes, and BMI).

The study was approved by the Institutional Ethics Review Board under protocol number 026/2013. Ethical principles involving research on human beings have been respected. The workers accepted to participate in the study after the objectives were clarified and they signed free and informed consent forms.

## 3. Results

The mean age of the 38 workers involved in the study was 50.16 years (SD ± 13.39 years). The majority of workers were female (*n* = 28; 73.7%), white (*n* = 34; 89.5%), and married (*n* = 31; 81.6%%) and were with incomplete elementary school (*n* = 30; 78.9%) (Table 1). The average time of rural work was 32.73 years (SD ± 21.73 years).

**Figure 1:**
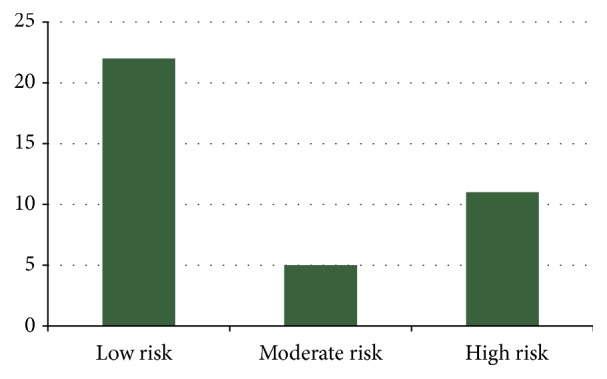
Global cardiovascular risk classification in 10 years of rural workers in the extreme south of Brazil.

The global cardiovascular risk classification in 10 years using the GRS showed a predominance of low risk (*n* = 22; 57.9%). However, 11 rural workers (28.9%) had a high cardiovascular risk (Figure 1).

In the detailing of the GRS, the frequency of high overall cardiovascular risk in 10 years was higher in men aged between 60 and 74 years. Spearman's correlation analysis showed significance between GRS and sex (rho = 0.623, *p* ≤ 0.001), age (rho = 0.783, *p* ≤ 0.001), systolic blood pressure (rho = 0.545, *p* ≤ 0.001), and smoking (rho = 0.483, *p* = 0.002). Thus, males with more advanced age, high systolic blood pressure, and smokers present correlation with a higher GRS, which classifies them as being at greater overall cardiovascular risk in 10 years (Table 2).

## 4. Discussion

It is known that, to evaluate the cardiovascular risk of rural workers, the ideal would be to complement the data acquired with lipid values (HDL cholesterol and total cholesterol) [17, 18]. In the present study, the cardiovascular evaluation of rural workers through GRS occurred through the identification of BMI [11]. Due to this, the present study presents a tendency regarding the presence of cardiovascular risk in a group of rural workers, indicating a high cardiovascular risk for 28.9% of the workers studied. A study conducted in rural Uganda, Africa, found high cardiovascular risk in 42% of participants [19], which shows the importance of classifying the risk to which the rural populations are exposed.

In addition, the results point to components that may increase the risk for CVD, such as increased age, high systolic blood pressure, smoking, and high BMI. Statistical tests showed a positive correlation between GRS value and gender, age, systolic blood pressure, and smoking. Thus, males with more advanced age, high systolic blood pressure, and smokers are correlated with a higher GRS, which classifies them as being at a higher overall cardiovascular risk in 10 years.

Previous studies performed with a greater number of participants, the first with 903 and the second with 492 men and women, also pointed out such correlation [20, 21]. Both studies showed that increased BMI is related to increased hypertension. These indications suggest that high BMI may be acting as a link to increase systolic blood pressure and, consequently, increase cardiovascular risk in these individuals. Thus, it is important to work to reduce the BMI of individuals, not just rural workers, the focus of this study, but all individuals who are identified with this condition. An Australian study that determines the effect of weight reduction on cardiovascular risk has shown that reducing weight reduces atrial fibrillation and consequently reduces the risk of developing CVD [22].

Nonmodifiable characteristics such as age and gender also correlated with increased cardiovascular risk for rural workers. The WHO points out that CVD affects both men and women; however, men are affected earlier by the disease, about 7 to 10 years before women [1]. Thus, health professionals need to be aware of the factors that may indicate an increase in cardiovascular risk in men before women, mainly due to the difficulty of health access by these individuals, which increases more in the case of residents of the rural area [15], as in the case of individuals in this study.

Another important result is the correlation between increased cardiovascular risk and smoking. This relationship is widely reported in the literature, showing that there is a relationship between smoking and CVD, and the higher the consumption, the greater the cardiovascular risk and, consequently, the cessation is the best way to revert the damage and prevent major injuries, among them, cardiovascular damage [23, 24].

These results point to a trend of the profile of rural workers that needs direct care, since the change in behavioral habits, such as food reeducation and reduction of smoking, requires continuous attention by health professionals. It is necessary to search for cardiovascular risk reduction strategies, such as a health care program for individuals with a high cardiovascular risk for health status management and thus an improvement in the quality of health care of these individuals [25].

## 5. Conclusions

This study aimed to classify the cardiovascular evaluation of rural workers through GRS and to analyze the intensity of the relationship between the classification and the variables of the score. Eleven workers were identified as at high cardiovascular risk according to the GRS and workers with increased age and high systolic blood pressure, smokers, and those with high BMI were correlated with a high cardiovascular risk score. These results show that rural workers need attention with regard to components that may put them at risk for CVD. The components identified in this study are blood pressure, smoking, and obesity. Follow-up is necessary for this group of rural workers in the development of biochemical researches for the confirmation of biomarkers (cholesterol, HDL cholesterol, and glycemia) and subsequent update of cardiovascular risk.

The limitations of the study include the analysis model, which does not allow establishing associations about the GRS; however, it is visualized that through the analysis it was possible to stratify the GRS (low, moderate, or high) and to analyze the correlation between the increase of the score and the variables that compose it. Thus, the present study presents the beginning of a deepening occupational research in rural areas, more specifically, on CVD, and this may aid in the early diagnosis and more effective actions on the risk factors for these diseases.

It is important to say that all workers, regardless of whether they are at cardiovascular risk or not, have received guidelines for reducing components that contribute to increased cardiovascular risk.

## Figures and Tables

**Table 1:** Socioeconomic characterization of rural workers in the extreme south of Brazil (*n* = 38).

Variables	*n*	%
*Gender*		
Female	28	73.7
Male	10	26.3

*Skin color/ethnicity*		
White	34	89.5
Black	02	5.3
Asian	01	2.6
Indigenous	01	2.6

*Marital status*		
Married	31	81.6
Single	03	7.9
Separated	02	5.3
Widowers	02	5.3

*Schooling*		
Not literate	04	10.5
Elementary school, incomplete	30	78.9
Elementary school	02	5.3
Secondary school, incomplete	01	2.6
Secondary school	01	2.6

**Table 2:** Distribution of the variables of the global risk score of rural workers in the extreme south of Brazil (*n* = 38).

Variables	*n* (%)	Low risk *n* (%)	Moderate risk *n* (%)	High risk *n* (%)	Spearman correlation	*p* value
*Gender*						
Male	10 (26.3)	01 (2.6)	01 (2.6)	08 (21.1)	0.623	≤0.001
Female	28 (73.7)	21 (55.3)	04 (10.5)	03 (7.9)		

*Age*						
30–39 years	11 (28.9)	11 (28.9)	00 (0.0)	00 (0.0)		
40–49 years	06 (15.8)	04 (10.5)	01 (2.6)	01 (2.6)	0.783	≤0.001
50–59 years	09 (23.7)	04 (10.5)	02 (5.3)	03 (7.9)		
60–74 years	12 (31.6)	03 (7.9)	02 (5.3)	07 (18.4)		

*Systolic blood pressure*						
90–119 mmHg	11 (28.9)	10 (26.3)	00 (0.0)	01 (2.6)	0.545	≤0.001
120–149 mmHg	19 (50.0)	11 (28.9)	03 (7.9)	05 (13.2)		
150–180 mmHg	08 (21.1)	01 (2.6)	02 (5.3)	05 (13.2)		

*Treatment for hypertension*						
Yes	09 (23.7)	04 (10.5)	01 (2.6)	04 (10.5)	0.227	0.171
No	29 (76.3)	18 (47.4)	04 (10.5)	07 (18.4)		

*Smoker*						
Yes	11 (28.9)	03 (7.9)	01 (2.6)	07 (18.4)	0.483	0.002
No	27 (71.1)	19 (50.0)	04 (10.5)	04 (10.5)		

*Diabetes*						
Yes	04 (10.5)	01 (2.6)	00 (0.0)	03 (7.9)	0.243	0.141
No	34 (89.5)	21 (55.3)	05 (13.2)	08 (21.1)		

*Body mass index*						
18.50–24.99	11 (28.9)	08 (21.1)	00 (0.0)	03 (7.9)		
25.00–29.99	14 (36.8)	09 (23.7)	02 (5.3)	03 (7.9)	0.206	0.216
30.00–34.99	09 (23.7)	02 (5.3)	03 (7.9)	04 (10.5)		
35.00–39.99	03 (7.9)	02 (5.3)	00 (0.0)	01 (2.6)		
≥40.00	01 (2.6)	01 (2.6)	00 (0.0)	00 (0.0)		
